# Vitamin D: Newer Concepts of Its Metabolism and Function at the Basic and Clinical Level

**DOI:** 10.1210/jendso/bvz038

**Published:** 2020-02-08

**Authors:** Daniel D Bikle

**Affiliations:** Department of Medicine and Endocrine Research Unit, Veterans Affairs Medical Center and University of California, San Francisco, California

**Keywords:** metabolism, receptors, vitamin D binding protein, nutrition, clinical trials

## Abstract

The interest in vitamin D continues unabated with thousands of publications contributing to a vast and growing literature each year. It is widely recognized that the vitamin D receptor (VDR) and the enzymes that metabolize vitamin D are found in many cells, not just those involved with calcium and phosphate homeostasis. In this mini review I have focused primarily on recent studies that provide new insights into vitamin D metabolism, mechanisms of action, and clinical applications. In particular, I examine how mutations in vitamin D metabolizing enzymes—and new information on their regulation—links vitamin D metabolism into areas such as metabolism and diseases outside that of the musculoskeletal system. New information regarding the mechanisms governing the function of the VDR elucidates how this molecule can be so multifunctional in a cell-specific fashion. Clinically, the difficulty in determining vitamin D sufficiency for all groups is addressed, including a discussion of whether the standard measure of vitamin D sufficiency, total 25OHD (25 hydroxyvitamin) levels, may not be the best measure—at least by itself. Finally, several recent large clinical trials exploring the role of vitamin D supplementation in nonskeletal diseases are briefly reviewed, with an eye toward what questions they answered and what new questions they raised.

## 1. Introduction

If one enters the term “vitamin D” into the PubMed search engine, looking for articles published on this subject over the past 5 years, one finds 21 545 such publications, with no evidence of a decline. Clearly vitamin D remains a popular subject of study. As such, no review can do justice to the many studies that contribute to the field, and this review is no exception. This review will reflect my own bias as to what I find new and exciting, acknowledging that it will not be inclusive of the many advances in this field over the past few years. What this review will cover is our changing perceptions of vitamin D metabolism based on the discovery of mutations in key enzymes affecting such metabolism as well as new insights into their regulation, mechanisms of action—including new information on how the VDR works in collaboration with other transcription factors and coregulators in different cellular contexts—changing concepts in how vitamin D sufficiency should be defined and measured, and, finally, an overall view of what recent large clinical trials are or are not telling us about who should take vitamin D supplements and in what doses and schedules.

## 2. Vitamin D Metabolism

As is well known, vitamin D is produced in the skin from 7-dehydrocholesterol (7-DHC), hydroxylated first in the liver (and other tissues) to 25 hydroxyvitamin (25OHD), and then in the kidney (and other tissues) to 1,25 dihydroxyvitamin D (1,25(OH)_2_D), with subsequent catabolism of both 25OHD and 1,25(OH)_2_D to their 24 (and 23) hydroxy forms, 24,25(OH)_2_D and 1,24,25(OH)_3_D (or 1,23,25(OH)_3_D). However, what is new are insights gained into the regulation of these steps, including some surprises about the enzymes involved, which we will now cover.

### A. 7-Dehydrocholesterol Reductase (DHCR7)

Although the production of vitamin D from 7-DHC under the influence of sunlight (UVB) is a nonenzymatic step, the production of 7-DHC is not. Its synthesis in the skin is a step in the Kandutsch-Russell pathway. DHCR7 converts 7-DHC to cholesterol, so its activity dictates how much 7-DHC is available for vitamin D production [1]. This enzyme is found in a number of tissues other than the skin, including the brain, muscle, and heart [2]. Inactivating mutations of DHCR7 result in the Smith-Lemli-Opitz syndrome, a developmental disorder [3, 4]. These patients suffer primarily from the consequences of too little cholesterol, steroids, or bile acids, but they appear to be more sensitive to UVB light, and may present with higher serum 25OHD concentrations than normal subjects [5]. On the other hand, cats and dogs have increased expression of DHCR7, limiting the amount of 7-DHC in their skin and so limiting their capacity to make vitamin D [6]. In humans, polymorphisms in DHCR7 have been associated with either reduced [7, 8] or increased [9] 25OHD levels. The regulation of DHCR7 is incompletely understood. Cholesterol and vitamin D (but not 1,25(OH)_2_D) increase proteasomal degradation of DHCR7, leading to increased vitamin D production [10]. AMPK, a key sensor and regulator of cellular energy homeostasis and protein kinase A are portent inhibitors of DHCR7, whereas CaMKII has a lower inhibitory effect [1, 11].

### B. 25-Hydroxylases

The liver is the major if not sole source of 25OHD production from vitamin D. Numerous enzymes within both mitochondria and microsomes were found to have 25-hydroxylase activity. Initial studies suggested that CYP27A1, a mitochondrial enzyme with substantial homology to both CYP27B1 and CYP24A1 (the 1α and 24-hydroxylases, respectively), was the major 25-hydroxylase. But patients with inactivating mutations in this enzyme develop cerebrotendinous xanthomatosis with abnormal bile and cholesterol metabolism, but not rickets [12]. Thus, the major role of CYP27A1 is to convert cholesterol to cholic acid and chenodeoxycholic acids such that mutations in CYP27A1 lead to reduced bile acid production and increased cholestanol that accumulates in the brain, causing progressive neurologic dysfunction [13] Moreover, in mouse studies, deletion of the Cyp27a1 failed to lower blood levels of 25OHD (deletion actually led to increased levels) [14]. Current data support CYP2R1 as the major 25-hydroxylase at least in the liver (and testes), where it resides in the microsomal fraction. CYP2R1 25-hydroxylates both D_2_ and D_3_ with comparable kinetics, unlike CYP27A1. When deleted from mice, blood levels of 25OHD fall over 50%, but not to zero [14], with little impact on blood levels of calcium and phosphate [14], suggesting compensation by other enzymes with 25-hydroxylase activity. Five functional mutations in CYP2R1 have been described so far. A Nigerian with severe bone disease and biochemical evidence of rickets including a low 25OHD but normal 1,25(OH)_2_D was discovered to have a leu99pro mutation in CYP2R1, the first mutation identified [15]. When tested in vitro this mutation was shown to reduce 25-hydroxylase activity [15]. Additional mutations include K242N, found in a compound heterozygote with CYP2R1 deficiency and familial rickets [16], compound heterozygote mutations involving a splice site (367 + 1,G to A) and an insert (768,iT) [17] in an Arabian family, and a deletion (gly42_leu46 with an inserted arg) in a French family [18]. Although these mutations resulted in little or no 25-hydroxyase activity in vitro, the subjects maintain normal or even high 1,25(OH)_2_D levels, and in some cases respond to both vitamin D and 1αOHD with further increases in 1,25(OH)_2_D [16, 18]. As adults they tend to lose their need for vitamin D supplementation [18]. Such data suggest that as in the mouse, CYP2R1 is not the only enzyme with 25-hydroxylase activity; enzymes that may be upregulated to compensate when CYP2R1 activity is blunted.

CYP3A4, the major drug metabolizing enzyme preferentially located in the liver and intestine, may be one of these compensating enzymes, as it has 25-hydroxylase activity [19]. Complicating the story, however, is that CYP3A also degrades both 25OHD and 1,25(OH)_2_D, hydroxylating them in the 23 and 24 positions like CYP24A1 (to be discussed later) [20]. Moreover, an activating mutation in CYP3A has recently been reported that increases 1,25(OH)_2_D and likely 25OHD catabolism, resulting in decreases in these metabolite levels and rickets [21].

Previously, it had been thought that the 25-hydroxylation of vitamin D was primarily substrate dependent. However, recent evidence indicates that this is not the case. Roizen et al found that the serum concentration of 25OHD, but not vitamin D, was decreased in mice fed a high-fat diet to induce obesity compared with normal weight mice [22]. Moreover, mRNA and protein levels of CYP2R1 were decreased in these obese mice. The expression of other 25-hydroxylases (CYP27A1, CYP3A) or the catabolizing enzyme CYP24A1 was not altered. Aatsinki et al examined the effect of high-fat diet–induced obesity, fasting, and type 2 diabetes, as well as streptozotocin-induced (type 1) diabetes, on 25OHD levels in mice [23]. All these metabolic manipulations decreased the hepatic mRNA and protein concentration of CYP2R1. These authors then demonstrated that the decrease in CYP2R1 was mediated by PPARγ-coactivator-1α (PGC1α), a key metabolic regulator increased by fasting or diabetes. They then showed that the control of *CYP2R1* gene expression by PGC1α involved another transcriptional regulator, estrogen-related receptor α (ERRα), which also binds to other nuclear receptors such as VDR and the glucocorticoid receptor (GR). Consistent with this is that dexamethasone, a ligand for GR, also decreased hepatic CYP2R1 mRNA and protein concentrations by a mechanism mediated by increased PGC1α. Thus, our concept that the low levels of 25OHD in obesity are somehow related to an increased storage of vitamin D in fat needs to be reexamined.

### C. *CYP27B1—the 25OHD-1*α *Hydroxylase*

Unlike the 25-hydroxylases, there is only one 25OHD-1α hydroxylase described, CYP27B1. This enzyme is found in the mitochondrion along with CYP24A1. The kidney is the main source of circulating 1,25(OH)_2_D, but many tissues, including the epidermis and other epithelial tissues, bone, placenta, and cells of the immune system, also express CYP27B1, producing 1,25(OH)_2_D likely for paracrine or autocrine uses [24]. Regulation of CYP27B1 in these extracellular sites differs from that in the kidney. In the kidney CYP27B1 is regulated primarily by parathyroid hormone (PTH), which stimulates, and fibroblast growth factor 23 (FGF23) as well as 1,25(OH)_2_D itself, which inhibit, whereas cytokines such as interferon gamma (IFNγ), tumor growth factor alpha (TNFα), and transforming growth factor beta1 (TGFβ1) are the major inducers of CYP27B1 in nonrenal tissues such as keratinocytes and macrophages [25, 26]. Dexamethasone, on the other hand is suppressive [27, 28]—at least in macrophages. Moreover, in keratinocytes, activation of TLR2, but not TLR4 (by LPS), induces CYP27B1 [26]. In peripheral blood mononuclear cells IL-1, IL-2, and IL-15 also stimulate CYP27B1 activity, whereas IL-4 is suppressive [29, 30]. Thus, the induction of CYP27B1 in these extrarenal tissues by cytokines and the failure of CYP27B1 in these tissues to respond to the increased circulating levels of 1,25(OH)_2_D and calcium account for the hypercalcemia often found in granulomatous diseases such as sarcoidosis and lymphomas [24, 31]. Although CYP27B1 regulation has been best studied in keratinocytes and macrophages, tissues such as the parathyroid gland, placenta, and bone have their own set of regulators [32].

Mutations in CYP27B1 cause a disease known as pseudovitamin D deficiency rickets or vitamin D-dependent rickets, type 1A (VDDR1A) [33, 34]. These individuals are characterized by normal to high 25OHD levels and low but not always absent 1,25(OH)_2_D levels. A recent study demonstrated that mice with a global deletion of *Cyp27b1* are able to produce normal levels of 1,25(OH)_2_D when given large doses of 25OHD, suggesting that other 1 hydroxylases may exist, although none have yet been identified [35]. The sequence of the gene was subsequently determined [36–39], enabling the mutations causing this disease to be identified [38, 40]. Both the renal and extrarenal CYP27B1 have the same sequence, but their differences in regulation occur as a result of differences in tissue-specific multicomponent control modules within the regulatory regions of the gene [41]. These studies will be further described when the mechanism of action of VDR is addressed.

### D. CYP24A1 and CYP3A—the 25OHD-24 (23) Hydroxylases

These are the catabolic enzymes of vitamin D metabolism, with both 25OHD and 1,25(OH)_2_D as their substrates. In most tissues CYP24A1 is the dominant 24-hydroxylase, but CYP3A4 likely plays a role in the liver and intestines, where it is highly expressed. CYP3A4 lacks the specificity for vitamin D metabolites shown by CYP24A1 [20], but drugs like rifampin can increase its expression leading to osteomalacia [42]. Both enzymes have 24-hydroxylase and 23-hydroxylase activity, although the relative proportions of 24-hydroxylase and 23-hydroxylase activity for CYP24A1 is species-specific [43]. Both enzymes are induced by 1,25(OH)_2_D, and the induction of CYP3A4 seems to be at least as great as that for CYP24A1 in the intestine [44]. The 24-hydroxylase pathway terminates with the biologically inactive calcitroic acid, whereas the 23-hydroxylase pathway produces the biologically active 1,25,26,23 lactone. These multistep reactions are all catalyzed by one enzyme, CYP24A1 [45]. To label CYP24A1 as a purely catabolic enzyme in vitamin D metabolism is a misnomer. 1,24,25(OH)_3_D has a substantial affinity for the VDR, with biological activity approximately 10% of 1,25(OH)_2_D. Moreover, a specific receptor for 24,25(OH)_2_D, Fam57B2, has been identified in bone and other tissues such as the skin, and through this receptor 24,25(OH)_2_D was found to be involved in fracture repair [46]. Deletion of Cyp24a1 in mice results in marked decreases in bone mineralization comparable to osteomalacia, which is rescued by also deleting the VDR, leading the authors to attribute the changes to large increases in 1,25(OH)_2_D [47]. Whether this also applies to humans with biallelic mutations, which, as noted below, results in hypercalcemia with increased 1,25(OH)_2_D levels, has so far not been reported [48]. Polymorphisms of the CYP24A1 gene are responsible for modest genetic variability of serum 25OHD (CYP24A1 is one of the 8 genes known so far to result in genetically predisposed higher or lower serum 25OHD concentrations). CYP24A1 is under the control of 1,25(OH)_2_D and FGF23 (both stimulatory) and calcium [49]. 5α-dihydrotestosterone, via the progesterone receptor, has also been reported to stimulate CYP24A1 [50]. In humans, inactivating mutations in CYP24A1 is now recognized as a major cause of idiopathic infantile hypercalcemia (IIH), a syndrome marked by severe hypercalcemia, hypercalciuria, and nephrocalcinosis, decreased PTH, low 24,25(OH)_2_D, and inappropriately normal to high 1,25(OH)_2_D [51]. At this point no skeletal defects have been described. Twenty-one missense mutations have recently been reported in the *CYP24A1* gene [52]. Although initially identified in children, recent case reports indicate that the diagnosis may not be made until adulthood, generally following a condition of increased 1,25(OH)_2_D production like pregnancy [53, 54], although not always [48, 55]. Such adults generally present with early onset nephrolithiasis and/or nephrocalcinosis. It is possible that the hypercalcemia in some patients with granulomatous diseases or lymphomas may have an underlying defect in CYP24A1 activity and its failure to compensate for the increased 1,25(OH)_2_D production in these diseases.

### E. Cholesterol side chain cleavage enzyme (CYP11A1)

This enzyme is the rate-limiting enzyme in steroid synthesis, but studies from the Slominski laboratory [56] demonstrated that CYP11A1 also metabolizes vitamin D_3_ to 20(OH)D_3_, with subsequent metabolism to additional metabolites that have biologic activity comparable in some cases to 1,25(OH)_2_D_3_. CYP11A1 is expressed in the skin and cultured keratinocytes [57] as well as better known steroid-producing tissues such as the adrenals, ovary, testes, and placenta. At this point, little is known about how this enzyme is regulated in the skin and elsewhere with respect to its vitamin D metabolizing activity, and its relative importance in the physiology of vitamin D remains unclear.

## 3. Mechanisms of Action

The VDR is critical for most of the actions of vitamin D, with 1,25(OH)_2_D as its major ligand. Vitamin D receptor is a transcription factor found in nearly all cells, although to variable levels. It is a member of the steroid hormone receptor family. Although initially identified in the small intestine, the VDR has subsequently been found in essentially all tissues in which it has been sought [58, 59]. Not surprisingly, vitamin D impacts many cellular processes via the VDR. In a recent ontology analysis 11 031 putative VDR target genes were identified, of which 43% were involved with metabolism, 19% with cell and tissue morphology, 10% with cell junction and adhesion, 10% with differentiation and development, 9% with angiogenesis, and 5% with epithelial to mesenchymal transition [60]. Furthermore, VDR can regulate various miRNAs and long noncoding RNAs involving the expression of numerous proteins directly or indirectly [61–63]. As a result of the appreciation that the VDR is so widespread, along with the key vitamin D metabolizing enzymes such as CYP27B1 and CYP24A1 [64], interest in understanding the role of vitamin D and the VDR in nonclassic, as well as classic, target tissues regulating calcium and phosphate homeostasis has been substantial. Although most actions of VDR involve its role as a transcription factor within the nucleus [65–67], the VDR has also been shown to have nongenomic actions via its location in the membrane [68] and perhaps even in mitochondria [69].

### A. Structure

The human VDR is located in chromosome 12 and is comprised of 9 exons. Exon 2 contains the translation start site and the nucleotide sequence encoding the short A/B domain (24 amino acids), to which transcription factors such as TFIIB bind, and the first zinc finger of the DNA binding domain (DBD) (65 amino acids). Exon 3 encodes the second zinc finger of the DBD. Exons 4–6 encode the hinge region (143 amino acids). Exons 7–9 encode part of the hinge region, the entire ligand binding domain (E/F) (195 amino acids) including the AF2 domain, and the extensive 3 prime untranslated regions (UTR). A polymorphism (FokI restriction site) is found in the human VDR, changing an ATG to an ACG and shifting the translation start site by 3 amino acids, shifting the total length from 427 to 424 amino acids. Numerous other polymorphisms have been described in untranslated regions of the VDR gene, to which associations with various diseases have been made. Amino acids 49–50 between the 2 zinc fingers and 102–104 C-terminal to the zinc fingers appear to provide the nuclear localization signal [70, 71]. The DBD is comprised of 2 zinc fingers held in a tetrahedral configuration by 4 cysteine residues. The first zinc finger directs DNA binding in the major groove of the DNA binding site. The second zinc finger provides a dimerization interface for its primary partner RXR [72]. The ligand binding domain is comprised of 12 α helices (H1-12) [73]. The terminal H12 provides the mobile portion of the mousetrap model for binding of 1,25(OH)_2_D to the VDR in that in the nonliganded VDR H12 is open, but with ligand binding H12 moves over the ligand enclosing it in the ligand binding pocket [73]. In the closed position H12 along with H3 and H4 provide the interface for coactivators such as the steroid receptor coactivator (SRC) and mediator complexes, which bind to H12 via their nuclear box (NR) containing the LxxLL sequence of amino acids (L for leucine, X for any amino acid) [74]. The ligand binding pocket is actually quite large and accommodates a number of ligands, including lithocholic acid and the 1,25(OH)_2_D analog with two side chains called gemini [75]. H9 and H10 along with the second zinc finger provide the heterodimerization site for RXR [76]. The membrane VDR accepts a different configuration of 1,25(OH)_2_D (6-S cis rather than 6-S trans conformation), leading Mizwicki et al [77] to propose an alternative pocket for ligand binding that is not yet confirmed. Amino acid residues critical for the structure/function of the VDR have been well demonstrated by mutations in the VDR, either in the human or mouse gene (reviewed in [78]).

### B. Regulation

The regulation of VDR expression is cell specific. For example, 1,25(OH)_2_D autoregulates VDR expression in bone cells but not in the intestine [79, 80]. Many factors including 1,25(OH)_2_D regulate VDR expression, including growth factors such as FGF, EGF, IGF, insulin, as well as parathyroid hormone, glucocorticoids, estrogen, and retinoic acid in some cases acting via a variety of transcription factors such as AP-1, SP1, C/EBP, CDX2, C/EBPβ, Runx2, cyclic AMP response element binding protein (CREBP), retinoic acid receptor (RAR), and GR [81]. Similarly, calcium upregulates VDR expression in the parathyroid gland, presumably through its calcium sensing receptor [82]. On the other hand, SNAIL 1 and 2 (SLUG) down regulate VDR expression in a number of cancer cell lines [83, 84]. MiRNAs can regulate VDR levels as exemplified by the binding of miR-125b, miR-298, and miR-27b to the 3’UTR to decrease VDR levels [85–87]. The VDR promoter may also be hypermethylated by various methylases and methyl transferases reducing its expression [88].

### C. Genomic Actions

Carlberg [65] reported that the human genome contains over 23 000 VDR binding sites, most of which are cell-specific [89]. Their locations varied with duration of ligand exposure, and only some were readily identified with a specific gene [90]. Most but not all of these binding sites were ligand dependent [91]. Although the DR3 was the most common binding sequence, it occurred in only a minority of binding sites, albeit the ones with the highest affinity for VDR. No obvious consensus DNA sequences have emerged for most non-DR3 binding sites. The DR3 refers to a DNA sequence with 2 hexanucleotide half sites separated by a 3 nucleotide spacer, with the consensus sequence of the half sites as A/G G G/T T C/G A. The VDR binding sites can be thousands of bases away from the transcription start site (TSS) of the genes they regulate, and genes generally have multiple VDR binding sites, the activity of which may vary in different cells and different species. The likely explanation for this variability is that the DNA can be looped to bring the relevant regulatory elements adjacent to the TSS. This function is performed by CCCTC binding factors (CTCF), which define genomic insulator regions, although not all CTCF sites are involved with insulator actions. Genomic loops of hundreds to thousands of bases can be looped into topologically associating domains (TADs) organizing the genome into several thousand such domains [92]. An informative example of how this might work in different cells is the regulation of the *RANKL* gene (*Tnfsf11*). This gene is regulated by PTH and 1,25(OH)_ 2_D in osteoblasts but by AP-1 factors such as c-fos in activated T-cells. The Pike Laboratory identified 7 VDR binding sites in *RANKL* up to 88kb upstream of the TSS, of which the -75kb site proved most active in the mouse gene [93, 94], whereas the proximal site was most active in the human gene [95]. However, in activated T-cells, 3 additional sites even further upstream of the TSS have been identified as sites of *RANKL* induction by c-fos [96]. CTCF binding sites separate the *RANKL* gene from adjacent genes, but also separate the VDR binding sites in osteoblasts from the more upstream c-fos binding site active in T-cells, potentially enabling different looping in these 2 types of cells [66, 96]. A similar example can be found for *Cyp27b1*. This gene is negatively regulated by its product in the kidney, but not in other tissues [97]. Pike et al identified a specific enhancer module that mediates endocrine regulation of the *Cyp27b1* gene in the kidney but not in nonrenal cells [41]. They then deleted this kidney-specific module regulating *Cyp27b1* to create a kidney-specific *Cyp27b1* pseudo-null mouse [98]. Subsequently, they also identified chromatin-based mechanisms involved in the differential regulation of Cyp24a1 in kidney and nonrenal target tissues [99]. The VDR binding sites are generally situated in a region with other transcription factors that may share regulation of that gene, potentially providing cell-specific regulation of the gene. For example, the VDR binding region of the *RANKL* gene contains several CREB sites responsible for PTH regulation of this gene [100, 101]. Runx2 and C/EBPβ binding sites can frequently be found in the same regions as the VDR binding sites and influence VDR activity [102]. Vitamin D receptor binding sites have also been colocalized with C/EBPβ, AP-1, CDX2, vTCF4 [103], ras activated Ets transcription factors [104], and YY1 [105, 106]. Changes in VDR binding sites, as can occur during differentiation of the cell, can affect the binding of these other transcription factors in a similar fashion [107, 108] as may account for the ability of 1,25(OH)_2_D to inhibit the early stages of osteoblast differentiation while promoting the later stages [109] or the sequential effects of 1,25(OH)_2_D on gene expression during keratinocyte differentiation [110].

### D. Coregulators and Epigenetic Changes Regulating VDR Function

The sites of active transcription are marked by epigenetic changes in both the gene itself and the histones that regulate access of the transcriptional machinery to the gene (review in [111]). For example, H3K4me3 (histone 3, in which lysine 4 is triply methylated) is found in sites of active transcription, whereas H3K9 is associated with silent promoter regions. H3K27ac is associated with active enhancers, whereas H3K27me is associated with gene suppression. 1,25(OH)_2_D can increase or decrease the expression of these methylases [112]. Sites of epigenetic gene suppression are also generally marked by methylation of cytosine in CpG islands (review in [113]) in association with the epigenetic changes in the histones listed above [114]. 1,25(OH)_2_D regulates these epigenetic changes by affecting the binding of coregulators to the VDR, whether coactivators with histone acetyltransferase activity (HATs) or cosuppressors with histone deacetylase activity (HDACs). There are over 250 published coregulators interacting with nuclear hormone receptors [115]. The best studied coactivators with respect to the VDR are the steroid hormone receptor coactivators (SRC 1–3) [116] and the Mediator complex [117]. Phosphorylation of serine 208 in the VDR following dimerization with RXR enhances their binding [118, 119]. The SRCs have 3 NR boxes containing the LxxLL motif described earlier to which they bind the nuclear hormone receptors [120], but VDR binding is primarily to the 3^rd^ NR box [121]. Med 1, the principal component of the Mediator complex binding to VDR and other nuclear hormone receptors, has 2 NR boxes, the second NR box being the one binding to VDR [121]. Steroid coactivator receptors recruit HATs to the VDR, in particular CREB binding protein CBP/p300 [122] and CBP/p300 associated factor p/CAF [123]. The Med complex does not contain HAT activity but binds directly to RNA polymerase II to help form the preinitiation complex along with basal transcription factors such as TFIIB and several TAT binding proteins [124]. The peroxisome proliferator-activated γ coactivator-1α (PGC-1α) described earlier in relationship to Cyp2r1 regulation interacts with VDR to regulate genes involved with muscle development and energy metabolism [125]. These coactivators all bind to the AF2 domain (H12). NCoA62/SKIP does not bind to the AF2 domain but binds to the VDR/RXR heterodimer to increase transcription with SRC but could also coordinate with corepressors NCoR/SMRT to suppress transcription [126, 127]. The best studied corepressors are the SMRT and NCoR complexes [128, 129], as they have HDAC activity. These corepressors bind to nuclear hormone receptors via their CoRNR boxes, which are analogous to the NR boxes of the SRC family but with a sequence LxxH/IxxxI/L (L for leucine, H for histidine, I for isoleucine) [130]. These corepressors do not bind to the AF2 domain (H12) but to H3-H5 in the absence of ligands. In the presence of 1,25(OH)_2_D and the conformational change with H12, these corepressors are displaced, enabling the coactivators to bind to their sites on H12. Hairless is a corepressor of VDR expressed primarily in the brain and skin [131]. It binds to the central region of the LBD of VDR, as do NCoR/SMRT to two ϕxxϕϕ hydrophobic sites (ϕ = hydrophobic aa) [132] but also to H12 via its LxxLL motif, which is similar to SRC and Med1 [121]. The role of hairless is complex in that it represses ligand-dependent VDR functions with respect to epidermal differentiation [131] but is required for ligand independent VDR regulation of hair follicle cycling [133].

### E. Nongenomic Actions

1,25(OH)_2_D also exerts rapid effects on cells that involve nongenomic mechanisms. Rapid increases in intestinal calcium transport following 1,25(OH)_2_D administration was the first such example, called transcaltachia [134], but similar effects have been seen in a number of other cells, including chondrocytes, osteoblasts, keratinocytes, and fibroblasts. Pathways involved in these rapid effects of 1,25(OH)_2_D include phospholipase C (PLC), phospholipase A_2_ (PLA), phosphatidylinositol-3-kinase (PI3K), wnt5a, and p21 ras, opening-up calcium and chloride channels with the generation of second messengers such as calcium, cyclic AMP, fatty acids, phosphatidylinositol 3,4,5 triphosphate (IP3) activating downstream protein kinases A and C, src, mitogen-activated protein kinases (MAPK), and calmodulin kinase II (reviews in [135, 136]). Two receptors for 1,25(OH)_2_D, both in the membrane, have been identified as mediating these rapid actions. The first such receptor initially called membrane-associated rapid response steroid (MARRS) binding protein [137] also goes by the names of thioredoxin-like protein (GRP58), endoplasmic reticulum protein 57/60 (ERp57 or 60), and protein disulfide isomerase family A, member 3 (Pdia3) [138]. The second receptor is the VDR itself, but in its membrane location it is activated by 1,25(OH)_2_D analogues with a different configuration (6-S cis) than those that activate the genomic actions of VDR (6-S trans), as mentioned earlier [139, 140]. These receptors can co-localize with caveolin-1 in caveolae [141] and may physically interact [142]. Similarly, interaction between the membrane VDR and the nuclear VDR has been suggested. For example, in kidney and intestinal cells the rapid activation of ERK 1/2, JNK1/2, PKC, PI3K, and p21ras have all been shown to regulate 1,25(OH)_2_D induction of Cyp24a1 [143–145] by a number of mechanisms, including phosphorylation of RXRα and Ets-1. The VDR may alter the actions of other transcription factors in a nongenomic fashion, as exemplified by its interaction with IкB kinase, to block NFкB signaling [146]. Similarly, MARRS/ERp57/PIA3 interacts directly with NFкB to facilitate the translocation of the complex into the nucleus to promote the differentiation of NB4 leukemia cells [147]. Deletion of MARRS/ERp57/PIA3 is embryonic lethal [148], but the heterozygote and the chondrocyte-specific deletion of MARRS/ERp57/PIA3 result in an enlargement of the growth plate, with an increased hypertrophic zone, increased apoptosis of the chondrocytes, and decreased trabecular bone [148, 149]. This phenotype has been attributed to increased endoplasmic reticulum (ER) stress due to improper protein folding in the ER because of the loss of this protein [149]. These findings disappear following puberty [149]. Thus, the mechanisms of VDR can be quite complex and generally cell specific with numerous layers of regulation.

## 4. Vitamin D Sufficiency

25OHD is the principal measure by which vitamin D sufficiency is currently determined for several reasons. First, 25OHD is the vitamin D metabolite in highest concentration in the blood, so it is the easiest to measure. Second, nearly all 25OHD is in the blood, unlike vitamin D itself, which is stored in tissues such as fat. Third, 25OHD has a half-life in blood measured in weeks, unlike hours for 1,25(OH)_2_D. That said, total 25OHD may not be the best measurement of vitamin D status.

### A. Free Versus Total 25OHD

25OHD, like other vitamin D metabolites, is carried in blood by 2 major proteins, vitamin D binding protein (DBP) and albumin. D binding protein binds whereas albumin binds 15% of the total 25OHD approximately 0.03% free in normal individuals [150]. Except for tissues expressing the megalin/cubilin complex, it is the free fraction that is generally considered to enter most cells (the free hormone hypothesis).This is similar to that for the thyroid and steroid hormones [151, 152], which like vitamin D metabolites are transported in blood bound to their respective binding proteins. There are a number of drugs and cytokines (dexamethasone and IL-6, for example) as well as clinical conditions (eg, pregnancy, liver disease, nephrotic syndrome, primary hyperparathyroidism, and acute trauma) that influence the circulating levels of DBP, effectively altering the ratio of total to free 25OHD [153, 154]. Thus, measuring just the total 25OHD levels would be misleading if in fact it was the free concentration to which most cells respond. Therefore, the determination of the free level of 25OHD might be a better, or at least a complementary, assessment of vitamin D status than the total level, although this remains controversial [154, 155].

### B. Assay Concerns

An additional consideration in determining vitamin D sufficiency is the variability among assays [156]. Most currently used assays involve antibodies that may differ between laboratories in their ability to measure 25OHD_2_ compared to 25OHD_3_ or their sensitivity to interfering substances [157]. The increasing use of mass spectroscopy coupled with an initial chromatographic step (LC/MS/MS) to measure vitamin D metabolites ameliorates many of the problems with immunometric assays [157], although this method remains restricted to larger clinical and commercial laboratories. Meanwhile, efforts are being made to harmonize the data from different laboratories in different countries [158] so that recommendations can be based on data comparable from one study to another. The Vitamin D Standardization Program (VDSP) was developed in an attempt to rectify this situation [159]. The VDSP coordinates the international effort to standardize serum 25OHD measurement to gold standard reference assays that have been developed. To this end the National Institute of Standards and Technology (NIST) has developed reference methods and materials for standardizing the measurements of a number of vitamin D metabolites. Finally, even if the problems with the assays are solved, it is not clear that 25OHD levels that are optimal for Caucasians are the same as those for Black Africans, Hispanics, or Asians. Not surprisingly, therefore, current guidelines vary, and at this point current guidelines must be considered a work in progress.

### C. Current Guidelines

The most consistent data, and the data used in establishing recommendations either for optimal levels of circulating 25OHD levels or levels of dietary supplements, come from studies involving the musculoskeletal system. In the various guidelines provided by different agencies different terms are used, such as the estimated average requirement (EAR), which is the median level at which 50% of the population would be sufficient; the required daily requirement (RDA), which is the level that meets the needs of 97.5% of the population; or terms such as adequate intake (AI), reference nutrient intake (RNI), and recommended intake (RI). The recommended levels for dietary intake assume no additional contribution from epidermal production of vitamin D.

In an international effort to provide a consistent set of guidelines, 33 nominated experts from pediatric endocrinology, pediatrics, nutrition, epidemiology, public health, and health economics recently published their consensus recommendations on the prevention and management of nutritional rickets [160]. They defined vitamin D sufficiency as 25OHD levels above 50 nM (20 ng/ml), insufficiency between 30–50 nM, and deficiency below 30 nM (12 ng/ml). Moreover, they noted that the consequences with respect to the development of rickets/osteomalacia were also dependent on calcium intake. They defined calcium sufficiency as > 500 mg/day, insufficiency as 300 –500 mg/day, and deficiency as < 300 mg/day. Individuals with the combination of insufficient or deficient levels of either calcium or vitamin D and deficient levels of the other were most at risk for rickets. Deficiency of vitamin D with a normal calcium intake might lead to biochemical abnormalities but not to rickets/osteomalacia, according to their analysis. They recommended 400 IU of vitamin D/day for infants up to 1 year and followed the Institute of Medicine’s (IOM) (now known as the National Academy of Medicine [NAM]) recommendations for older children and adults (see below).They further recommended 200 mg calcium/day for infants up to 6 months, and 260 mg/day from 6–12 months. Recommendations for calcium intake of older children were 500 mg calcium/day or more. Other than the recommendation of the NAM for adults to ingest 700–1300 mg calcium/day [161], most other guidelines are silent with respect to calcium. With respect to vitamin D the NAM recommended 600 IU from years 1–70 and 800 IU above age 70. The NAM considered a 25OHD serum level of 50 nM (20 ng/ml) to suffice for 97.5% of the population [161]. The Endocrine Society guidelines recommend 75 nM (30 ng/ml), considering levels between 50–75 nM as insufficient and less than 50 nM as deficient [162]. Although the Endocrine Society’s recommendations for children and adults are comparable to other groups (400 IU/day for infants, 600 IU/day from ages 1–70, 800 IU/day for those over 70), the Endocrine Society suggests that doses up to 2000 IU of vitamin D/day may be necessary to achieve and maintain a serum 25OHD level of 75 nM (30 ng/ml). Guidelines from other countries have recently been reviewed [163] and in general are consistent with those described above.

Some guidelines also include upper limits beyond which more vitamin D and/or calcium is not recommended. The NAM, for example, recommends that vitamin D intake should not exceed 4000 IU/day or a calcium intake above 2000–3000 mg/day (based on age) [161]. However, other studies indicate that at least in healthy individuals, doses up to 10 000 IU/day of vitamin D are not toxic. The upper limits of calcium intake beyond which toxicity develops are less clear, as the doses recommended have also been associated with increased kidney stones and cardiovascular disease [164].

## 5. Clinical Applications

As noted above, the guidelines are based primarily on data from studies of the musculoskeletal system, both association studies and randomized clinical trials, as summarized in a recent review [165]. Baseline vitamin D and calcium levels appear to be important markers for responsiveness. A 400 IU dose of vitamin D sufficed to prevent rickets in a study in Turkey, where deficiency was extensive [166]. Other studies in adult populations demonstrated elevated PTH levels and reduced bone mineral density at 25OHD levels below 50 nM [167, 168] and increased osteoid suggestive of osteomalacia in hip fracture patients with 25OHD levels below 30 nM [169, 170]. At these levels vitamin D supplementation shows a clear benefit [171]. Vitamin D supplementation works better when used in conjunction with calcium [172–174], especially in the elderly and vitamin D deficient. Although not all studies have demonstrated protection against fractures, several have when elderly vitamin D deficient populations are studied [174, 175], but not when vitamin D sufficient populations are studied [176]. Moreover, the amount and administration schedule also matter. Use of very high doses of vitamin D (ie, 300 000–500 000 IU given annually) seems to increase fracture risk [177, 178]. Similar conclusions can be reached for falls. Daily doses of 700–1000 IU of vitamin D reduce the risk of falls in the elderly, especially those with vitamin D insufficiency [179, 180], but higher doses intermittently administered appear to increase the risk of falling [177, 181]. The reduction in falls is likely related to improvement in muscle function, as seen best in the most frail with lowest 25OHD levels [182]. With respect to nonmusculoskeletal diseases, although vitamin D supplementation has been associated with reduced overall mortality, most diseases that have shown an association with low 25OHD levels, with the exception of upper respiratory illnesses and asthma, have not yet shown consistent benefit with vitamin D supplementation [183, 184]. However, most of these studies have not discriminated between participants with sufficient versus insufficient 25OHD levels, so the jury is still out. Two recently published large clinical trials exemplify this point. The VITAL study [185] examined the ability of vitamin D, 2000 IU/day, and/or omega 3 fatty acid to reduce the risk of cancer and cardiovascular events. This was a very large trial, with 25 871 participants (men over 50, women over 55), including a large number of African Americans with a median study duration of 5.3 years. The median 25OHD level was 31 ng/ml, which is considered vitamin D sufficient even by Endocrine Society guidelines. The primary endpoints of invasive cancer and major cardiovascular events showed no significant risk reduction, although in a secondary analysis the group with a body mass index (BMI) of less than the median 27.1 taking vitamin D had a significant reduction in invasive cancer. This is of interest given that as discussed in the section on vitamin D metabolism, this group may have had a higher CYP2R1 level and so responded to the vitamin D supplementation with a greater increase in 25OHD. This remains to be demonstrated, as post-trial 25OHD levels were not determined. Moreover, it was not clear whether individuals with low 25OHD levels at baseline responded better to supplementation than those with normal levels of 25OHD. The D2D study [186] evaluated the ability of vitamin D, 4000 IU/day, to reduce the risk of developing diabetes mellitus in 2423 prediabetics over a mean time of 2.5 years. The median baseline 25OHD level in this population was 28 ng/ml. Although the hazard ratio in the vitamin D supplemented group was 0.88 relative to placebo controls, this was not quite statistically significant (*P* = 0 = .12), although in a post hoc analysis, subjects with baseline 25OHD levels < 12 ng/ml had a significant benefit from vitamin D supplementation (HR 0.38). Moreover, like the VITAL study, the group of study subjects with BMIs less than 30 also showed a significant reduction in progression to diabetes in a subgroup analysis. Therefore, we may expect that future clinical trials will focus on nonobese subjects with baseline 25OHD levels in the insufficient range, as they may have a greater response to the supplementation.

## 6. Conclusion

Studies in vitamin D over the past several years have produced a number of new insights into vitamin D metabolism, mechanisms of action, and guidance for clinical applications, as summarized in Table 1. Mutations in the metabolizing enzymes have helped elucidate the role of these enzymes in a number of clinical conditions. New discoveries into the regulation of these enzymes has shown important links between metabolic conditions and vitamin D metabolism. Better understanding of how the VDR interacts with other transcription factors in a cell-specific fashion provides greater understanding of how the same molecule can have such different actions in so many physiologic processes. New and better methods of measuring the vitamin D metabolites and an understanding of the role of DBP in regulating the total and free levels of these metabolites provides us a greater appreciation of what constitutes vitamin D sufficiency and the difficulties in reaching consensus of such. Total 25OHD levels may not be the best index of vitamin D sufficiency, and the level optimal for one group or one outcome may not be optimal for other groups or outcomes. It is not surprising then that recommended levels for vitamin D intake and optimal levels of circulating 25OHD levels vary somewhat from country to country and group to group. Clinical trials to address some of these questions have shown that vitamin D supplementation of individuals with normal 25OHD levels on average has a limited impact—at least over the short duration of the trials—on a number of nonskeletal conditions, but subpopulations such as the nonobese and those with low baseline levels of 25OHD may benefit. The answers are not all in, but the strides over the last few years have answered some of these questions, though they have also opened more.

**Table 1.** New Concepts in Vitamin D Metabolism and Function

• The level of 7-dehydrocholesterol, the substrate for vitamin D production, is controlled by the activity of 7-dehydrocholesterol reductase, an enzyme in the skin regulated by vitamin D and AMP kinase, thus linking vitamin D production to regulation other than that of UVB exposure.
• The metabolism of vitamin D to 25OHD by the main 25-hydroxylase CYP2R1 is regulated by metabolic conditions, including obesity.
• CYP3A4 like CYP24A1 metabolizes 25OHD and 1,25(OH)2D in both the 23 and 24 positions and may play a major role in vitamin D catabolism in the liver and intestines. Drugs like rifampin that induce CYP3A4 can lead to osteomalacia.
• CYP27B1 regulation differs in different tissues because of differences in access of regulatory factors to enhancer modules in the CYP27B1 gene secondary to differences in chromatin organization.
• Mice lacking Cyp27b1 if given enough 25OHD will normalize their 1,25(OH)2D levels, suggesting other enzymes with 1α-hydroxylase activity.
• Tissue specificity in vitamin D function relates to differences in the regulation of VDR levels within different tissues and the tissue specific array of ancillary transcription factors and coregulators.
• The “non genomic” 1,25(OH)_2_D binding protein variably known as MARRS/ERp57/PIA3 when deleted is embryonic lethal, but when deleted specifically in chondrocytes or in heterozygotes results in an abnormal growth plate and decreased bone formation that reverses after puberty.
• Vitamin D sufficiency may be better characterized by measuring both the total and free concentrations of 25OHD.
• Large randomized clinical studies of subjects with normal vitamin D at baseline have shown little benefit from vitamin D supplementation.

## Additional Information

***Disclosure Summary:*** The author has nothing to disclose.
